# Blood Stream Infections

**DOI:** 10.1155/2014/515273

**Published:** 2014-07-01

**Authors:** Renu Bharadwaj, Abhijit Bal, Ketoki Kapila, Vidya Mave, Amita Gupta

**Affiliations:** ^1^Department of Microbiology, BJ Government Medical College and Sassoon Hospital, Pune 411001, India; ^2^Department of Medical Microbiology, University Hospital Crosshouse, NHS Ayrshire & Arran, School of Medicine, University of Glasgow, Glasgow G12 8QQ, UK; ^3^Department of Microbiology, Armed Forces Medical College, Pune 411040, India; ^4^Johns Hopkins University India Center, BJ Medical College, Pune 411001, India; ^5^Center for Clinical Global Health Education, Division of Infectious Diseases, Johns Hopkins School of Medicine, 600 North Wolfe Street, Phipps 540B, Baltimore, MD 21287, USA

Blood stream infection (BSI) is one of the most devastating preventable complications in Critical Care Units. It has far-reaching consequences resulting in prolonged length of hospital-stay, high costs to the individual and exchequer, and, in many instances, loss of life. Although exact rates of BSI differ markedly worldwide, figures in the US are around 19.8 episodes per 1000 central-line days (CI 95%; 16.1–23.6) with an approximate incidence of 100,000 episodes annually [1]; the rate, in USA, falls to 5.8 (CI 95%; 3.8–7.8) when only microbiologically documented episodes are considered [2] and to 8.75% in Indian ICUs [3]. The need to bring these two data groups (microbiologically proven and clinical sepsis) as close as possible is every infectious disease specialist's dream.

Advances in medical science have resulted in increased interventions in critically ill patients creating foci from where bacteria can gain access to the blood stream resulting in an increase nosocomial BSI. They represent about 15% of all nosocomial infections and affect approximately 1% of all hospitalized patients [1]. A hospital-related BSI would deem to have occurred after a patient has completed ≥48 h of stay in the hospital or has a central line for 48 h or more [4]. Community acquired BSIs can also occur. A BSI is primary when the central line is the only probable source of infection and secondary when there is an underlying cause for the BSI (genitourinary/respiratory infection or any other obvious source of infection in the body).

Among the bacterial causes of BSI,* Staphylococcus aureus*, coagulase negative* Staphylococci*, and* Enterococcus faecalis* are the commonest among Gram positive organisms;* Escherichia coli*,* Klebsiella pneumonia*, and* Serratia spp* are the commonest among Enterobacteriaceae; and* Pseudomonas spp* and* Acinetobacter baumannii* are the commonest amongst the nonfermenter Gram negative organisms [2, 5]. Among fungi, it is nonalbicans* Candida* spp followed by* Candida albicans* that are common [6]. However, organisms vary with several factors such as (i) type of health-care facility involved, (ii) presence of a central venous/arterial catheter, (iii) type of catheter used, (iv) duration of catheterization, (v) prevalent organisms in the center, (vi) immune status of the host, (vii) underlying comorbidities, (viii) level of preventive and barrier precautions undertaken, and (ix) initial antimicrobial therapy [1, 7].

The severely immune-compromised patient is prone to fungal as well as bacterial blood stream infections. However, the lack of diagnostic tools for early detection of candidemia and other fungal infections limits the number of studies on this issue. Clinical and radiological signs are nonspecific, and traditional culture-based tools suffer from low sensitivity. Tests that have generated interest include combined detection of mannan and anti-mannan antibodies, *β*-1, 3, D-glucan detection, and a number of molecular techniques. Unfortunately drawbacks of nonculture techniques include moderate level of sensitivity and specificity and lack of standardization [8].

Currently, multidrug resistant bacteria, residing in ecological niches in hospitals, present therapeutic challenges when they manifest as bacteremias [7]. Bacteriological profile and drug resistance patterns tend to be peculiar to an institute that is dealing with a special category of patients. In this issue, a tertiary care center in Brazil reports a retrospective cohort study of the prevalence of vancomycin resistant* Enterococcus faecium* from BSI, and K. Gohel et al. report the profile and drug resistance patterns of blood culture isolates from a tertiary nephrourology institute of India. In India, the burden of bacterial infection is estimated to be very high; however, systematic data is limited [2]. Identification of the extent of the problem generates evidence for advocacy for regulation of currently unregulated antibiotics. Additionally, such data guides the policy on implementation of antibiotic stewardship programs and standardized infection control guidelines. Knowledge of the pattern of antibiotic resistance prevalent in severe infections could also motivate and direct new drug discovery.

The need for early aggressive therapy in BSI cannot be overemphasized. The “time-window” for administration of appropriate therapy is <6 h once symptoms are apparent and many agree that the very first hour is critical [9]. An ideal platform must offer quick, specific diagnosis, be economical, and have minimum hands-on time. Use of biomarkers for diagnosis and monitoring of sepsis holds promise. Not only do they distinguish infective from noninfective sepsis, but also the serial use of biomarkers can be used for determining effectiveness of an intervention. Procalcitonin and C-reactive protein are already in use widely but the search is on for even better agents. The performance of soluble triggering receptor expressed on myeloid cells-I (sTREM-1), soluble urokinase-type plasminogen receptor (suPAR), proadrenomedullin (pro ADM), and presepsin appears promising and offers better prognostic performance than procalcitonin [10, 11]. The use of biomarkers in sepsis has been discussed in this special issue. Apart from biomarkers, a mass-spectroscopy based approach known as MALDI-TOF (Matrix-assisted laser desorption/ionization time-of-flight mass spectrometry) can provide genus and species level identification within minutes enabling significant time saving over conventional methods of identification [12]. Research is on to determine the potential of mass-spectrometry to provide other useful information to the clinician, epidemiologists, and clinical microbiologists, such as genotyping, virulence marker, and resistance mechanism. A modified PCR/ESI-MS (PCR followed by electrospray ionization mass spectrometry) method is now available and holds promise for detection of pathogen directly from clinical samples. This evolving method has been discussed in this special issue. The need of the hour for the technology sector is to work upon such tools that are not only efficient but also economically viable so that developing nations can benefit as, ironically, it is here that the need for such measures is most.

The therapeutic challenges posed by the blood stream pathogens make it imperative that better strategies are developed to prevent infections. Education and training of health-care workers, use of maximum sterile barrier precautions for all patients on central/peripheral lines, and appropriate skin antisepsis during central venous catheter insertions are some simple guidelines that can save precious lives [12]. Medicated catheter-lock solutions and use of antiseptic/antibiotic impregnated central venous catheter and chlorhexidine impregnated sponge dressings are being looked into in various centers [1, 4].

This special issue brings forth various aspects of blood stream infections from around the world, including advances in detection and use of possible alternative pharmaceutical agents. The quest for reducing blood stream infections is gaining momentum worldwide as in most cases, it is eminently preventable. BSI that plagues critical care centers all over the world is a continued challenge, has many formidable frontiers, and remains an enigma even today. We hope this issue will stimulate researchers to work on improving the methodologies for detection, prevention, and management of blood stream infections so that we can reach a stage of “zero” morbidity and mortality from this infection.

*Renu Bharadwaj* *Abhijit Bal* *Ketoki Kapila* *Vidya Mave* *Amita Gupta*
